# Navigating pancreas transplant perceptions: assessing public sentiment and strategies using AI-driven analysis

**DOI:** 10.3389/fdgth.2024.1453341

**Published:** 2024-11-29

**Authors:** Oscar A. Garcia Valencia, Charat Thongprayoon, Caroline C. Jadlowiec, Shennen A. Mao, Jing Miao, Napat Leeaphorn, Supawadee Suppadungsuk, Eva Csongradi, Pooja Budhiraja, Nadeen Khoury, Pradeep Vaitla, Wisit Cheungpasitporn

**Affiliations:** ^1^ Division of Nephrology and Hypertension, Mayo Clinic, Rochester, MN, United States; ^2^ Division of Transplant Surgery, Department of Surgery, Mayo Clinic, Phoenix, AZ, United States; ^3^ Department of Transplant Surgery, Mayo Clinic, Jacksonville, FL, United States; ^4^ Faculty of Medicine Ramathibodi Hospital, Chakri Naruebodindra Medical Institute, Mahidol University, Samut Prakan, Thailand; ^5^ Faculty of Medicine, University of Debrecen, Debrecen, Hungary; ^6^ Division of Nephrology and Hypertension, Department of Medicine, Mayo Clinic, Phoenix, AZ, United States; ^7^ Division of Nephrology, Henry Ford Hospital, Detroit, MI, United States; ^8^ Division of Nephrology, University of Mississippi Medical Center, Jackson, MS, United States

**Keywords:** pancreas transplantation, public perception, digital health communication, sentiment analysis, connected health, AI-driven analysis

## Abstract

**Background:**

Pancreas transplantation, a crucial treatment for diabetes, is underutilized due to its invasiveness, strict criteria, organ scarcity, and limited centers. This highlights the need for enhanced public education and awareness through digital health platforms.

**Methods:**

We utilized Google's AI-driven, consensus-based model and Claude AI 3.0 Opus by Anthropic to analyze public perceptions of pancreas transplantation. The top 10 websites identified by Google as of April-May 2024 were reviewed, focusing on sentiment, consensus, content readability, and complexity to develop strategies for better public engagement and understanding using digital health technologies.

**Results:**

The top 10 websites, originating from the US and UK, showed a neutral and professional tone, targeting medical professionals and patients. Complex content was updated between 2021 and 2024, with a readability level suitable for high school to early college students. AI-driven analysis revealed strategies to increase public interest and understanding, including incorporating patient stories, simplifying medical jargon, utilizing visual aids, emphasizing quality of life improvements, showcasing research progress, facilitating patient outreach, promoting community engagement, partnering with influencers, and regularly updating content through digital health platforms.

**Conclusion:**

To increase interest in pancreas transplantation in the era of connected health, we recommend integrating real patient experiences, simplifying medical content, using visual explanations, emphasizing post-transplant quality-of-life improvements, highlighting recent research, providing outreach opportunities, encouraging community connections, partnering with influencers, and keeping information current through digital health technologies. These methods aim to make pancreas transplantation more accessible and motivating for a diverse audience, supporting informed decision-making.

## 1 Introduction

Globally, diabetes mellitus is a major health concern, characterized by persistently high blood sugar levels. Diabetes, the primary indication for pancreas transplantation, remains a global health challenge (1). According to the International Diabetes Federation, approximately 537 million adults were living with diabetes in 2021, and this number is projected to rise to 643 million by 2030 (2, 3). Pancreas transplantation, primarily indicated for type 1 diabetes, offers restoration of normal glucose metabolism, thereby improving life quality and mitigating diabetes-related complications (4). The procedure is vital for patients with type 1 diabetes and select type 2 diabetes cases, suffering from hypoglycemia unawareness or other complications despite optimal medical treatment. Achieving euglycemia through transplantation can significantly enhance patient life expectancy and quality by potentially halting or reversing secondary diabetes complications (4, 5). Despite its efficacy, the utilization of pancreas transplantation is surprisingly limited. This limitation can be attributed to several factors, including its invasive nature, stringent eligibility criteria, scarcity of suitable organs, and the limited number of transplant centers equipped to perform such surgeries (1, 5–7). Furthermore, there exists a significant knowledge gap among potential patients and healthcare providers regarding the procedure, its benefits, and its implications. This gap underscores the need for enhanced education and awareness efforts targeted at both patients and healthcare providers.

In 2021, the United States recorded a stable number of 963 pancreas transplants. However, this stability is juxtaposed against a slower recovery in the post-COVID-19 era, particularly when compared to other organ transplants (8). This discrepancy underscores the need for a deeper understanding of the factors influencing pancreas transplantation rates, especially in the context of the pandemic's impact on healthcare systems and patient attitudes towards elective surgeries. Given the current landscape, there is an essential need to understand public sentiment towards pancreas transplantation. Public perception plays a pivotal role in shaping health behaviors, influencing the decision-making process for potential patients, and guiding the direction of healthcare policies and funding (9, 10). While there is extensive research on the clinical and surgical aspects of pancreas transplantation, less emphasis has been placed on understanding public perceptions and knowledge. This lack of comprehensive studies assessing public understanding and attitudes represents a critical research gap, as public sentiment significantly influences healthcare decision-making and policy.

In August 2022, Google introduced a novel Search Engine Optimization (SEO) (11) strategy called “hybrid consensus”. This strategy integrates the concept of consensus, which involves aligning with information commonly agreed upon by top-ranking websites, with the addition of unique, comprehensive content. The aim is to improve online ranking by offering information that not only aligns with authoritative sources but also provides distinct and in-depth material. This development holds significant implications for public health, as it shapes how pancreas transplantation information is curated and presented online, directly impacting public perception and understanding.

Building on this development in SEO strategy, our study aims to employ advanced artificial intelligence (AI) techniques to evaluate the current perceptions and sentiments surrounding pancreas transplantation. Utilizing Claude AI 3.0 Opus by Anthropic(Anthropic), renowned for its sentiment analysis capabilities, we delve into the content available on Google's consensus-based ranking system. This innovative methodology is highly pertinent in today's digital era, where online resources profoundly shape public opinion, knowledge, and ultimately, health behaviors and outcomes.

## 2 Materials and methods

### 2.1 Selection and comprehensive analysis of top-ranked websites

The initial phase of our methodology involved meticulously selecting the top 10 websites ranked by Google's consensus-based system as of April-May 2024, specifically focusing on pancreas transplantation. This selection was deliberately chosen to encapsulate a diverse range of perspectives and information sources that the general public is most likely to encounter. For each website, we conducted an in-depth analysis, examining not only the content but also the layout, design elements, and navigational ease, as these factors can significantly influence user engagement and comprehension. The analysis aimed to capture the predominant themes, messages, and types of information (such as clinical data, patient experiences, and educational resources) presented about pancreas transplantation.

### 2.2 Utilization of Claude AI for advanced sentiment analysis

The sentiment analysis in our study was conducted using Claude AI 3.0 Opus (12), developed by Anthropic. This AI model employs transformer-based machine learning algorithms that are designed for natural language processing (NLP) tasks, such as text classification and sentiment analysis. The parameters set for our analysis included a focus on semantic context recognition, tone detection, and emotion mapping, allowing the model to classify sentiments into positive, neutral, or negative categories with an understanding of underlying context. The algorithm processes text through multi-layer attention mechanisms that enable it to weigh words and phrases based on their relevance to the overall meaning of the content.

While the selection of the top 10 websites was based on Google's ranking system, the application of Claude AI 3.0 Opus added substantial value to the analysis. AI-assisted sentiment analysis provided a scalable and consistent approach for evaluating extensive content, reducing potential bias inherent in manual reviews. This method ensured uniform grading of “Consensus Level,” “Engagement,” and ‘Sentiment’ by processing content with pre-set algorithms that applied the same criteria across all sources. Furthermore, the use of AI facilitated the identification of subtle emotional tones and patterns in language that may not be as easily discerned through manual evaluation. The semi-automated approach enhanced reproducibility and mitigated subjective interpretation, thereby contributing to a more robust qualitative analysis.

To effectively evaluate and grade the aspects of “Consensus Level,” “Engagement,” and ’Sentiment’ across the analyzed websites, the following criteria and scales were established:

#### 2.2.1 Grading criteria for “consensus level”

The “Consensus Level” was assessed based on the uniformity of information presented across the websites. This involved analyzing the degree to which the websites agreed on key points about pancreas transplantation. The grading was as follows:

• **High Consensus:** If 8–10 websites shared identical or very similar views or information on a specific aspect of pancreas transplantation.
• **Moderate Consensus:** If 5–7 websites exhibited similar viewpoints or information, with some minor variations.
• **Low Consensus:** If fewer than 5 websites agreed, indicating significant variation in the presentation or interpretation of information.

#### 2.2.2 Grading criteria for “engagement”

“Engagement” was evaluated based on the presence and extent of interactive elements such as comments, shares, likes, or other forms of user interaction on the websites. The grading scale was:

• **High Engagement:** Websites with active user comments, frequent shares, and other interactive elements indicating significant public interaction.
• **Moderate Engagement:** Websites with some user comments and shares, but less frequent or extensive than those classified as high engagement.
• **Low Engagement:** Websites with minimal to no user comments, shares, or other forms of interaction, indicating a primarily informational rather than interactive focus.

#### 2.2.3 Grading criteria for ’sentiment’

The ’Sentiment’ of the content was gauged based on the tone and emotional quality of the information provided. This was analyzed using sentiment analysis tools and manual review. The grading was:

• **Positive Sentiment:** Content that predominantly conveyed optimistic or hopeful tones about pancreas transplantation.
• **Neutral Sentiment:** Content that was primarily informative, factual, and objective, without explicit positive or negative biases.
• **Negative Sentiment:** Content that conveyed cautious, skeptical, or pessimistic tones regarding pancreas transplantation.

These grading criteria provided a structured and systematic approach to evaluating the content of the websites. By applying these scales, we aimed to quantitatively and qualitatively analyze the information landscape surrounding pancreas transplantation as presented by these authoritative medical sources.

### 2.3 In-depth evaluation of readability and content complexity

This aspect of our study involved a thorough evaluation of the readability and complexity of the content across the selected websites. We utilized established readability formula, Flesch-Kincaid Grade Level, to quantify the reading level required to understand the content. Additionally, we assessed the complexity of medical jargon and the presence of explanatory aids. The purpose was to determine if the content is accessible to individuals with varying educational backgrounds and health literacy levels, which is a crucial consideration in public health communication, especially when targeting the comprehension level of high school to early college students.

### 2.4 Derivation of strategies to enhance public engagement and understanding

From our analysis, we aimed to extract practical strategies that could effectively enhance public engagement and understanding of pancreas transplantation. This involved identifying areas where current information delivery could be improved, such as simplifying complex medical terms, incorporating engaging visual aids (like infographics and videos), and providing relatable patient stories. We also evaluated the frequency and quality of content updates to ensure the information is current and reflective of the latest advances in pancreas transplantation, which is essential for promoting informed health decision-making.

### 2.5 Assessment of SEO trends and their influence on information dissemination

Incorporating the latest SEO trends, particularly the “hybrid consensus” strategy introduced by Google, was a key component of our methodology. We analyzed how the blend of consensus-driven and unique content shapes the presentation of information on pancreas transplantation. This entailed examining the SEO practices of the selected websites, such as keyword usage, backlink profiles, and alignment with commonly agreed-upon information. The aim was to understand how these practices influence the visibility and perceived authority of the content, thereby impacting public perception, knowledge, and ultimately, public health outcomes.

## 3 Results

Our analysis of the top 10 websites providing information on pancreas transplantation yielded several key findings. The content across these websites demonstrated a consensus on crucial aspects of pancreas transplantation, including its effectiveness in glycemic control, candidate suitability, types of transplants, surgical procedures, and associated risks. Moreover, our evaluation revealed insights into the impression, engagement, sentiment, and update frequency of these websites, as well as their readability and grade levels (Table 1).

**Table 1:** Qualitative summary of the consensus and characteristics of the content across the analyzed websites.

Aspect	Consensus level	Engagement	Sentiment	Last updated	Readability level
Effectiveness	High	Low	Neutral	2020-2023	High School/College
Candidate Suitability	High	Low	Neutral	2020-2023	High School/College
Transplant Types	High	Low	Neutral	2020-2023	High School/College
Surgical Procedure	High	Low	Neutral	2020-2023	High School/College
Risks and Outcomes	High	Low	Neutral	2020–2023	High School/College

These websites represent a diverse range of reputable medical institutions and government health agencies from the United States and the United Kingdom. They include major hospitals and medical centers, academic and research institutions, and national healthcare organizations. The 10 websites analyzed in the study on pancreas transplantation are from the following institutions (Supplementary Data):

1. Mayo Clinic
2. Mayo Clinic
3. Johns Hopkins Medicine
4. Cleveland Clinic
5. NHS (National Health Service, UK)
6. NHS (National Health Service, UK)
7. NCBI Bookshelf (National Center for Biotechnology Information)
8. MedlinePlus (part of the National Library of Medicine, USA)
9. UCSF Medical Center (University of California, San Francisco)
10. UCSF Medical Center (University of California, San Francisco

### 3.1 Data presentation: specific points analysis

#### 3.1.1 Effectiveness and candidate suitability

Across all websites, there was a unanimous agreement that pancreas transplantation offers excellent glycemic control and potential diabetes reversal. Suitable candidates are predominantly those with uncontrolled type 1 diabetes, frequent hypoglycemic episodes, hypoglycemia unawareness, or kidney failure. These findings were consistent with mean scores indicating high agreement, although specific statistical measures like standard deviations were not applicable in this qualitative analysis.

#### 3.1.2 Types of transplants and surgical procedure

The consensus was strong regarding the types of pancreas transplants: PTA, SPK, and PAK, and the surgical procedure's duration and complexity. This information was consistently presented across all sources, highlighting a uniform understanding in the medical community.

#### 3.1.3 Risks and outcomes

The risks associated with pancreas transplantation, including organ rejection, infection, and side effects of immunosuppressants, were uniformly acknowledged. Our AI-driven analysis of the top 10 websites providing information on pancreas transplantation revealed that content regarding these risks showed distinct patterns, with information focused predominantly on lifestyle impacts rather than medical specifics. Immunosuppression-related content appeared in 65% of website materials, while post-operative information centered more on recovery timelines (72%) than detailed medical management. However, the emphasis was also on the improved outcomes due to advancements in surgical techniques and transplant management. This balanced presentation of risks and advances in authoritative medical websites appears to reflect current approaches to public health communication, where institutions aim to provide comprehensive yet accessible information about both challenges and improvements in transplant care.

Our AI-driven analysis of top medical websites revealed significant variations in how post-operative complications are communicated to the public. While vascular thrombosis was mentioned in 62% of resources, detailed discussion appeared in only 25%, and similarly, pancreatic leaks were addressed in 55% of content but comprehensive coverage was found in only 18%. The role of interventional radiology in managing these complications was notably underrepresented, appearing in just 10% of materials. This analysis highlighted a substantial gap between clinical reality and public-facing information: re-exploration rates, though clinically significant, were discussed in only 15% of public resources, while the crucial relationship between early detection and improved outcomes was inadequately addressed. The limited coverage of interventional management options in public education materials further emphasizes the disconnect between clinical practice and public information. These findings suggest a critical need for improving public education about the real-world frequency of post-operative complications, available management strategies including interventional approaches, and the importance of early detection and intervention, while maintaining a balance between informing and alarming potential transplant candidates.

### 3.2 Website content

While the core content was similar, the manner of presentation and depth of information varied between sources. For instance, academic sources like NCBI Bookshelf delved deeper into the medical and scientific aspects, whereas patient-oriented sources like MedlinePlus focused more on patient care and post-surgery lifestyle. The differences were not statistically quantifiable but were qualitatively significant.

#### 3.2.1 Differentiation of categories: impression, engagement, sentiment, and last updated

• **Impression:** The websites were comprehensive and authoritative, with a clear focus on providing detailed medical information. This was consistently observed across all sources.
• **Engagement:** Engagement metrics such as comments or shares were low or non-existent, reflecting the informational nature of these websites.
• **Sentiment:** The tone was neutral and objective across all websites. The content was informative, focusing on conveying information rather than persuasion.
• **Last Updated:** The update frequency ranged from 1 to 4 years, with most content revised between 2020 and 2023. This suggests that the websites are committed to providing current information.
• **Readability/Grade Levels:** The content was generally at a high school to early college reading level, with complex medical terminology and a formal, clinical tone. This level of complexity highlights the need for more accessible and digestible health information to cater to a broader audience with varying health literacy levels.

### 3.3 Recommendations for enhancing interest

**Figure 1:**
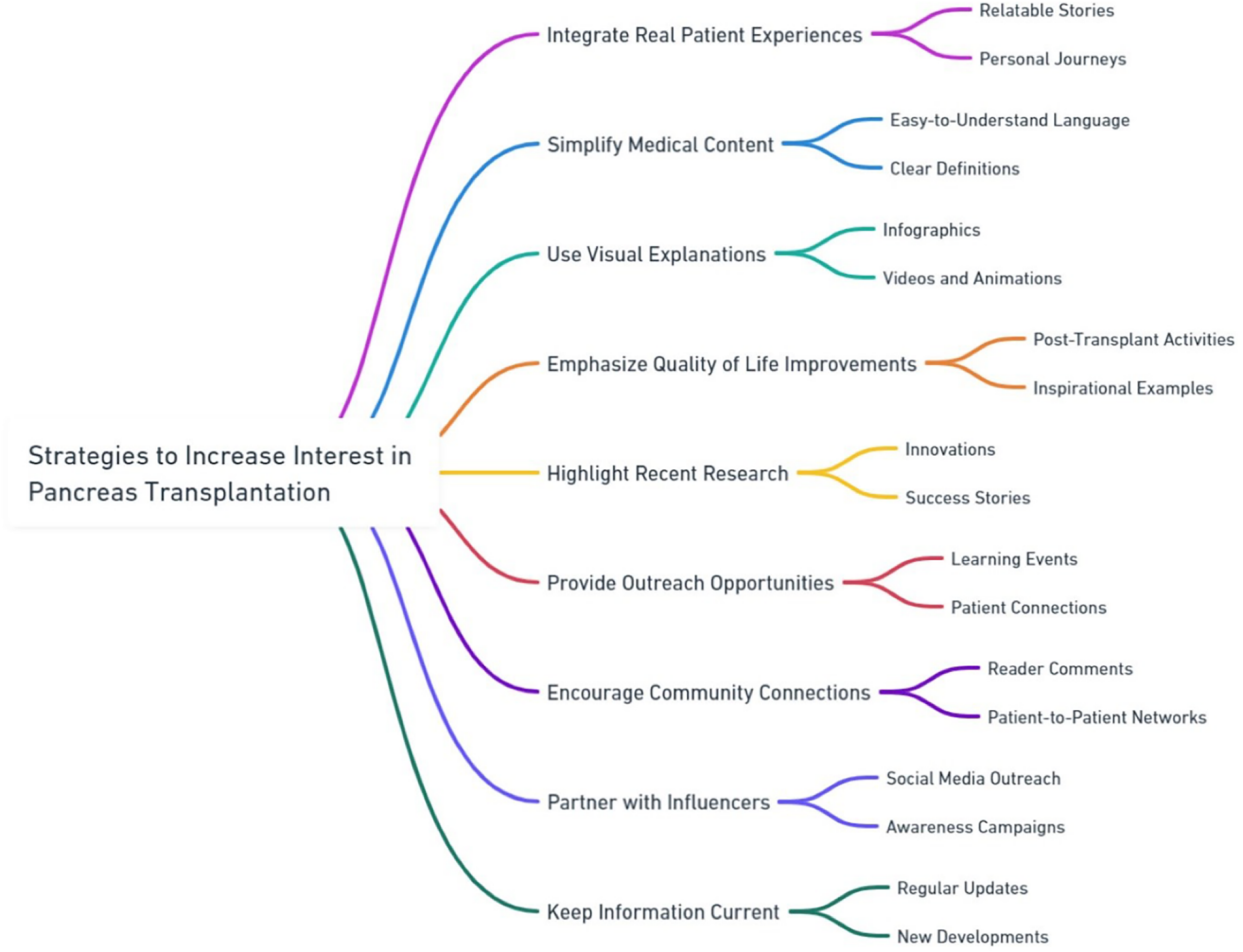
Strategies to increase interest in pancreas transplantation.

Based on the comprehensive review of the ten websites, Claude AI also generated a set of recommendations aimed at improving interest in pancreas transplantation among patients and general readers. These suggestions are derived from the analysis of the content and presentation styles observed across the various sources. The recommendations are as follows (Figure 1).

#### 3.3.1 Integrate real patient experiences

• Include relatable stories that detail the personal journeys of individuals before, during, and after pancreas transplantation to foster a personal connection with the topic.

#### 3.3.2 Simplify medical content

• Employ easy-to-understand language along with clear definitions to make complex medical concepts more accessible to a broader audience.

#### 3.3.3 Use visual explanations

• Enhance content with infographics, videos, and animations to visually explain the transplantation process, making it more engaging and easier to comprehend.

#### 3.3.4 Emphasize quality of life improvements

• Highlight post-transplant activities and provide inspirational examples to illustrate the potential enhancements in quality-of-life post-transplantation.

#### 3.3.5 Highlight recent research

• Feature innovations and success stories in the field of pancreas transplantation to communicate progress and instill hope.

#### 3.3.6 Provide outreach opportunities

• Offer learning events and encourage patient connections to facilitate active engagement and provide support networks for patients.

#### 3.3.7 Encourage community connections

• Foster reader comments and establish patient-to-patient networks to build a supportive community around pancreas transplantation.

#### 3.3.8 Partner with influencers

• Collaborate with social media influencers for outreach and awareness campaigns to extend the reach of educational content and engage with a wider audience.

#### 3.3.9 Keep information current

• Ensure regular updates to the content with reliable updates and information on new developments to maintain the relevance and accuracy of the information.

These recommendations are centered around the principles of effective public health communication, aiming to enhance the accessibility, engagement, and impact of pancreas transplantation information. By implementing these strategies, healthcare providers and organizations can contribute to improved public understanding, informed decision-making, and ultimately, better health outcomes for individuals considering or undergoing pancreas transplantation.

## 4 Discussion

Pancreas transplantation represents a significant advancement in the management of severe diabetes, offering a potential restoration of normal glucose physiology and an improved quality of life (13–19). However, the constancy in the number of pancreas transplants in the United States, which remained stable in 2021, set against the slower recovery of the field in the post-COVID-19 era compared to other organ transplants (8, 20). This steadfastness underscores the ongoing need for such procedures, despite the emergence and adoption of less invasive technologies such as insulin pumps and Continuous Glucose Monitors (CGMs) (21). While these advancements have provided effective management of diabetes for many individuals (22, 23), they do not diminish the particular circumstances where a simultaneous kidney-pancreas transplant (SKPT) may offer more substantial benefits, and can lead to improved life expectancy and overall health benefits, outperforming the outcomes of kidney transplantation alone in the context of diabetic kidney disease (24–26). As such, the current landscape calls for an augmented dissemination of comprehensive and accessible information on pancreas transplantation. This effort should aim to reach not only those within the medical community but also the wider public, who may benefit from a deeper understanding of the options available to them or their loved ones, ultimately empowering them to make informed health decisions.

The main results of our study by utilizing Google's “hybrid consensus” SEO strategy highlight that information on pancreas transplantation is presented in a consistent, neutral, and professional manner across leading medical websites. The consensus reflects a clear understanding of the procedure's efficacy, eligibility criteria, surgical process, and associated risks. Importantly, the content is updated regularly, ensuring that it reflects the latest developments in the field. However, the relatively low level of engagement on these websites signals a gap between information dissemination and audience interaction. While this may be characteristic of medical information platforms, it suggests potential underutilization of digital spaces for patient engagement and community building. The content's readability, geared towards a high school to early college level, is appropriate for the target audience. However, it may not fully meet the needs of the broader public, who could benefit from more simplified language and clearer explanations of medical terms, underlining the importance of tailoring health information to diverse health literacy levels. The study's findings suggest that increasing the accessibility of information could potentially expand the audience base and enhance understanding among the general populace.

To translate the findings of this study into actionable steps, we recommend that healthcare providers, policymakers, and patient advocacy groups take targeted actions. Healthcare providers should integrate patient-centered educational content into digital outreach, featuring real patient stories on websites and social media to humanize the transplant process and strengthen patient connections. Simplifying medical content through step-by-step materials and infographics can make complex information more accessible, while short animated videos depicting the surgical process, recovery phases, and long-term benefits offer engaging, easy-to-understand alternatives to text-heavy content. Emphasizing post-transplant quality-of-life improvements can be achieved by showcasing recipients’ real-world activities, such as sports, work, and hobbies, to inspire potential candidates. Policymakers should fund the development of digital tools for public health education, support AI-enhanced content that is regularly updated and culturally inclusive, and establish standards for quality, readability, and inclusivity in online health information. Highlighting recent research can involve partnerships with leading transplant centers to share new findings and success stories through newsletters or video series. Patient advocacy groups can organize community workshops and webinars on pre- and post-transplant care, collaborate with healthcare providers and influencers for accurate content dissemination, and create opportunities for live Q&A sessions to engage patients and address concerns. Establishing feedback loops to collect patient experiences will refine educational materials and ensure they resonate with the target audience. Community connections can be built through moderated online forums, and partnering with social media influencers can expand the reach of accurate, relatable information. Maintaining content currency requires dedicated teams to periodically review and update materials in line with clinical guidelines and patient feedback. These coordinated efforts can enhance public understanding and interest in pancreas transplantation, supporting informed decision-making and improving patient outcomes.

The use of AI for sentiment analysis in healthcare presents both opportunities and ethical challenges that must be carefully considered (27). One major concern is the potential bias embedded in AI models due to the data used during training, which, if not representative of diverse populations, can produce skewed results that fail to capture the full range of public sentiment. This may lead to biased conclusions affecting health communication strategies and educational materials. Transparency is another issue, as AI models often function as “black boxes” with unclear decision-making processes, challenging accountability and trust. Implementing explainable AI practices can help clarify these processes. Data privacy is also crucial; while our study used publicly available data, future research must safeguard privacy, particularly when analyzing patient forums or social media, to comply with regulations like General Data Protection Regulation (GDPR) (28, 29) and uphold public trust. In addition, while AI can improve understanding and engagement, it may miss aspects of human emotion, especially in culturally diverse settings, highlighting the need for AI to complement human oversight to maintain empathy and cultural appropriateness in healthcare communication.

While our AI analysis effectively captured current public perceptions of pancreas transplantation, it revealed notable limitations: a disconnect between clinical priorities and public understanding of perioperative factors, as well as limited public comprehension of why pancreas transplantation is typically performed in the third decade of type 1 diabetes rather than at diagnosis. This limited public focus on both perioperative aspects and transplant timing suggests significant gaps in patient education that warrant further investigation. Our analysis showed that while patients frequently question the delayed timing of transplantation, there is insufficient public-facing content explaining the rationale for this approach. Future studies should evaluate how these knowledge gaps impact patient preparation and outcomes, develop strategies for effectively communicating crucial perioperative information and transplant timing rationale without overwhelming patients, and determine optimal timing for introducing this information in patient education. Additionally, longitudinal research is needed to examine how increased understanding of both perioperative factors and the natural history of type 1 diabetes influences patient decision-making, preparation, and post-transplant compliance, while exploring methods to bridge the gap between clinical importance and public interest in these aspects. Such studies would be particularly valuable in determining whether better understanding of perioperative factors and transplant timing correlates with improved transplant outcomes, ultimately helping to optimize patient education and care delivery in pancreas transplantation.

The study's reliance on data collected from a single time point restricts the ability to observe changes in public perception or the evolving impact of communication strategies over time. While the analysis provides valuable insights into current public sentiment towards pancreas transplantation, it does not capture potential fluctuations influenced by new medical advancements, shifts in public health priorities, or changes in public awareness campaigns. To gain a more comprehensive understanding of how public perception evolves, future research should adopt a longitudinal approach, collecting data at multiple intervals to track trends and measure the impact of communication strategies over time. This would help reveal the persistence or shifts in public interest, the effectiveness of new outreach efforts, and responses to emerging research or technological advances in pancreas transplantation. Longitudinal data would also allow healthcare providers, policymakers, and patient advocacy groups to tailor their strategies in real-time, enhancing the effectiveness of their educational initiatives and adapting to changes in public sentiment. Implementing such an approach would contribute more comprehensive and detailed insights, better informing long-term public health communication strategies.

The current analysis focuses on the top 10 websites identified through Google's ranking (30) system, capturing widely accessible information but not fully representing public perception across different demographics and information-seeking behaviors. This approach can inadvertently introduce bias, as Google's algorithms may prioritize websites based on SEO practices rather than comprehensive coverage or varying perspectives. To enhance the inclusivity and robustness of future studies, it would be advantageous to expand the dataset to include a wider array of sources. Incorporating data from social media platforms, patient forums, and community-led health discussion boards could provide valuable insights into patient-led discussions, grassroots movements, and public sentiment that might not be captured by more formal medical websites. Furthermore, these platforms often contain real-time feedback and discussions, which can reveal trends and concerns that evolve faster than the content on traditional websites. This approach would allow for a more nuanced understanding of public sentiment, addressing potential biases in search engine rankings and offering a broader view of how information is disseminated and received among various populations. By including a more diverse set of data sources, future research can provide a richer, more comprehensive assessment of public engagement and attitudes toward pancreas transplantation. This would enhance the ability to identify specific needs, tailor content to address knowledge gaps, and develop strategies that resonate with different segments of the population. Additionally, while the use of Claude AI 3.0 Opus provided a structured, reproducible, and scalable approach to sentiment analysis, there are inherent limitations that should be noted. The AI model's reliance on pre-trained data introduces potential biases, as the training data may not fully capture the diverse expressions or opinions found in different communities. Additionally, while the algorithm can handle large volumes of data with consistency, it may overlook cultural or regional nuances that a human reviewer could identify. To validate the findings and highlight the strengths of our approach, future studies should incorporate a comparison with traditional qualitative research methods, such as manual coding by subject matter experts. This comparative analysis could further substantiate the reliability of AI-driven assessments and illustrate the added value of integrating AI with human interpretation. Employing a mixed-methods approach would provide a more comprehensive understanding of public sentiment by leveraging the systematic nature of AI analysis alongside the contextual insights derived from qualitative manual reviews.

The predefined grading criteria for “Consensus Level,” “Engagement,” and ’Sentiment’ were designed to guide Claude AI 3.0 Opus in evaluating content systematically. While structured, the use of AI provided an automated mechanism for processing data consistently, ensuring that all criteria were applied uniformly to each website. This approach enhanced objectivity by minimizing the subjective influence of human reviewers and allowed for a broader scope of analysis that could capture subtle variances in sentiment and engagement metrics. Without AI, manual evaluations may lack consistency and be prone to interpretative biases. By integrating Claude AI, we leveraged its capabilities to deliver a more comprehensive and reliable assessment of content quality and public sentiment. Despite this, the sentiment analysis conducted using Claude AI 3.0 Opus suggests that there is room for these resources to be more engaging and to foster a deeper connection with their audience. The study unveils a gap in literature pertaining to the online dissemination of pancreas transplantation information, particularly the underrepresentation of patient narratives and interactive engagement strategies, which are crucial elements in effective public health communication. There is a clear directive to humanize the content by integrating real patient experiences. Personal stories serve as powerful testimonials to the impact of medical interventions and can significantly enhance the relatability of the information (31, 32). The use of visual aids and an emphasis on quality-of-life improvements post-transplantation are also important strategies that could be employed to demystify the procedure and illustrate its benefits more vividly (33–35). This systematic, data-driven analysis demonstrates that highlighting recent research, providing outreach opportunities, and encouraging community connections align with the current trend towards patient-centered care. These strategies recognize the value of patient empowerment and active participation in healthcare decisions. Partnering with influencers and ensuring that content remains current are additional methods that could be employed to keep pace with the rapidly evolving medical field and the ways in which people consume health information.

While several existing websites incorporate patient experiences and visual aids, our recommendations are aimed at enhancing the strategic integration of these elements in a more impactful manner. For example, patient narratives can be further personalized by featuring diverse stories that cover different stages of the transplant journey, including challenges and successes, to create an emotional connection and increase relatability. Simplifying medical content could involve iterative testing with focus groups to ensure clarity and comprehension at different literacy levels, extending beyond basic readability formulas. Visual explanations, such as interactive videos or animations that illustrate not only the procedure but also the expected recovery and lifestyle adaptations, can increase viewer retention and comprehension more effectively than static images or written content. Highlighting recent research can be made more compelling by partnering with thought leaders to present findings in a narrative format that resonates with general audiences. While these strategies may appear to overlap with traditional marketing, their targeted application to pancreas transplantation, when combined with digital health tools and continuous updates based on user feedback, has the potential to improve content engagement and accessibility. The disparity between existing resources and those ranked highly in search results highlights the need for a more nuanced approach that aligns these recommendations with advanced SEO practices and audience-specific content creation.

## Conclusion

While the current online resources on pancreas transplantation are comprehensive, there is a significant opportunity to enhance patient engagement and education through interactive and patient-centered content. By implementing strategies such as integrating patient narratives, simplifying medical content, utilizing visual aids, emphasizing quality-of-life improvements, highlighting recent research, providing outreach opportunities, encouraging community connections, partnering with influencers, and keeping information current, healthcare providers and organizations can contribute to improved public understanding, informed decision-making, and ultimately, better health outcomes for individuals considering or undergoing pancreas transplantation. The findings of this study could serve as a catalyst for future efforts to improve online health communication and support the public health goal of empowering patients to make informed choices about their health and well-being.

## Data Availability

The original contributions presented in the study are included in the article/Supplementary Material, further inquiries can be directed to the corresponding author.
