# Supplementary material for: Navigating pancreas transplant perceptions: assessing public sentiment and strategies using AI-driven analysis
Source: Front Digit Health. 2024 Nov 29;6:1453341. doi: 10.3389/fdgth.2024.1453341 (PMC11638235; doi:10.3389/fdgth.2024.1453341)
Supplement: Supplementary file 1 [file Datasheet1.docx]

Supplementary Material

# Supplementary Data

**Website 1:** <https://www.mayoclinic.org/tests-procedures/pancreas-transplant/about/pac-20384783>

**Website 2** <https://www.mayoclinic.org/departments-centers/pancreas-transplant/sections/overview/ovc-20205138>

**Website 3** <https://www.hopkinsmedicine.org/health/treatment-tests-and-therapies/pancreas-transplant>

**Website 4.** <https://my.clevelandclinic.org/health/treatments/24384-pancreas-transplant>

**Website 5.** <https://www.nhs.uk/conditions/pancreas-transplant/>

**Website 6.** <https://www.nhs.uk/conditions/pancreas-transplant/why-its-done/>

**Website 7.** <https://www.ncbi.nlm.nih.gov/books/NBK562338/>

**Website 8.** <https://www.ucsfhealth.org/treatments/pancreas-transplant>

**Website 9.** <https://transplantsurgery.ucsf.edu/conditions--procedures/pancreas-transplant.aspx>

**Website 10.** <https://medlineplus.gov/ency/article/003007.htm>
